# A data privacy and deep learning based AMR dashboard for rural and regional veterinary practices in Texas

**DOI:** 10.3389/fvets.2025.1646675

**Published:** 2025-12-10

**Authors:** Saroj Gopali, Tianxi Ji, Marcelo Schmidt, Nancy Zimmerman, Babafela Awosile

**Affiliations:** 1 Department of Computer Science, Texas Tech University, Lubbock, TX, United States; 2 Texas Tech University School of Veterinary Medicine, Amarillo, TX, United States

**Keywords:** antimicrobial resistance (AMR), data privacy, deep learning, fingerprinting, Long Short-Term Memory (LSTM), Prophet, time series

## Abstract

Antimicrobial resistance (AMR) grows as a major worldwide health threat which affects treatment of both human and veterinary medicine. Practitioners working in rural and regional veterinary offices throughout Texas face difficulties obtaining real-time data tools which support making antimicrobial treatment decisions. This study introduces an AI-driven dashboard to address veterinary medicine needs by utilizing real-world AMR data collected over 14 years from veterinary labs throughout Texas. The dashboard employs deep learning models along with Long Short-Term Memory (LSTM) and Prophet with Recurrent Neural Networks (RNN) for prediction tasks and data imputation so practitioners can access insights utilizing visual elements such as heatmaps, Sankey plots, and MIC distributions and susceptibility tables. The dashboard empowers veterinarians with predictive analytics to perform empirical treatment selection and monitor resistance patterns to improve antimicrobial stewardship. Additionally, the dashboard integrates privacy-preserving fingerprinting techniques using steganographic marks, ensuring secure data sharing without compromising utility. Our novel approach addresses critical gaps in veterinary AMR data analysis, supporting antimicrobial stewardship and public health efforts through One Health frameworks. The findings demonstrate AI has proven its capacity to transform evidence-based veterinary medicine through data integrity and privacy.

## 1 Introduction

The global health community faces substantial challenges from antimicrobial resistance (AMR) since this phenomenon affects human medicine alongside veterinary medicine. The improper prescription and excessive use of antimicrobial drugs by veterinarians enables resistant microorganisms to develop which creates difficulties in treating animal infections and creates public health risks because these pathogens can transmit between animals and humans.

Current analysis of veterinary AMR data relies on traditional statistical approaches that fail to properly process the substantial amounts and intricate nature of data obtained from veterinary practices. Standard analysis techniques present drawbacks during large data set processing and pattern recognition which limit their usefulness in speedy decision support within veterinary practice. The present gap exists because clinicians need tools that unite different data types, including antimicrobial susceptibility test findings and patient information, along with epidemiological records for making effective antimicrobial therapy selections.

Artificial intelligence (AI) has emerged as a transformative technology in data analysis across various fields, including medicine. In veterinary medicine, AI applications are being explored for their potential to analyze complex datasets, predict disease outcomes, and optimize treatment strategies. Specifically, AI-driven approaches can predict the likelihood of resistance in specific pathogens and recommend the most effective antimicrobial agents, potentially improving outcomes and promoting antimicrobial stewardship.

The establishment of an automated AMR dashboard through AI faces difficulties because of mandatory reliable data systems as well as ethical AI implementation decisions and usability needs for veterinary staff. The system offers major potential to enhance antimicrobial stewardship efforts while addressing AMR challenges because it serves to improve animal health and public health safety.

The proposed study delivers essential contributions to veterinary medicine alongside antimicrobial resistance (AMR) data management because it investigates Texas's distinct geographical location while studying various animal species. Texas maintains an extensive rural and regional territory which creates an ideal setting for AMR research across livestock and companion animals. The dashboard targets the unique characteristics of Texas by collecting and employing antimicrobial sensitivity information from veterinary diagnostic labs operating within both rural and regional veterinary service areas across Texas. This initiative will use the collected antimicrobial susceptibility data to develop a regional dashboard that enables better empirical antimicrobial choices for rural and regional veterinary practice.

It also provides tracking of AMR trends together with future data predictions. This specially designed method ensures the dashboard includes features that address Texas rural veterinary practice needs despite their constrained resources and limited access to sophisticated tools. The main value of this research stems from its assessment of security and privacy in AMR data management since earlier works have neglected this fundamental aspect. Security measures for the proprietary dataset assume crucial importance since it holds sensitive data obtained from veterinary practices and diagnostic laboratories. The main contributions of the study are as follows:

- This paper is the first study to use real-world AMR data^1^ from rural veterinary practices in West Texas, covering 14 years (2011–2024), to support decision-making and analyze the developing AMR trends.
- The development of an AMR dashboard^2^ using antimicrobial susceptibility data from Texas's rural practices and labs will be developed via AI using deep learning models including LSTM and Prophet combined with features that include Sankey plots and heatmaps, MIC (Minimum Inhibitory Concentration) distribution, detailed reports and susceptibility tables alongside bacteria distribution trend tracking and empirical treatment selection support.
- This research will become the primary study to combine secure and private data management of AMR through innovative fingerprinting methods while protecting confidential information and still optimizing administrative decision quality supported by reinforced AI analytics.
- The dashboard implements Recurrent Neural Networks (RNN) for advanced data processing followed by the provision of a public dashboard with statistical testing along with educational resources for comprehensive AMR analysis of imputed datasets.
- The solution delivers broad benefits by supporting antimicrobial stewardship of Texas veterinary schools and rural practitioners and farmers along with pet owners through a public dashboard based on deep learning for actionable insights and evidence-based practice promotion.

Existing AMR dashboards either lack AI integration or do not support secure data sharing when resources are limited. To the best of our knowledge, this is the first AI dashboard that uses real data, together with forecasting and privacy-preserving. This paper fills the gap by offering a fully operational, explainable, and secure AI pipeline specifically designed for veterinary decision support.

The next sections of this paper follow a particular structure. Section 2 examines existing research in the field. Data and deep learning model, methodology of AMR dashboard appear within Section 3. Section 4 presents the analysis and visual outputs of the AMR Dashboard. Section 5 presents the veterinary insights. Section 6 describes the approach of data sharing. The paper ends with a conclusion and future works in Section 7.

## 2 Related works

The veterinary field adopts AI technology to evaluate sophisticated data along with forecasting results and enhancing therapeutic strategies. The research by Akinsulie et al. (1) demonstrates how artificial intelligence can track resistant bacteria as well as track antibiotic usage trends so our dashboard's objectives become achievable. The prediction of AMR uses Machine learning techniques as described by Caneschi et al. (4) which supports clinical practice decision-making.

Antimicrobial resistance (AMR) is a serious threat to global healthcare because it influences both human medical practices and veterinary therapeutic requirements. The improper usage and excess application of antimicrobial drugs in veterinary medicine have led to the development of resistant pathogens that makes animal infection treatment more complex and introduces potential infections from animals into human populations. Under the One Health framework which illustrates human-animal-environmental interconnectedness, multiple studies confirm the importance of this issue including Antimicrobial Resistance in Veterinary Medicine (3). The Use of Antibiotics and Antimicrobial Resistance in Veterinary Medicine demonstrates how antibiotics usage generates economic effects and healthcare consequences which require innovative solutions for crisis management (4). Standard statistical analysis techniques currently rule data analysis related to AMR in veterinary practice but struggle to manage comprehensive and extensive modern veterinary data sets. Present methods struggle to efficiently process enormous datasets while detecting fine patterns or generating prognosis data thus reducing their worth for live operational choices. A tool gap exists because veterinary practices need resources to combine antimicrobial susceptibility testing results with patient records and epidemiological information to allow clinicians more suitable antimicrobial selections.

AI has become an influential transformative technology that controls data analysis across all sectors including medical practices. Medical professionals are testing AI applications in veterinary medicine due to their ability to handle complex data and forecast disease outcomes and deliver optimal treatment plans. The potential application of artificial intelligence in veterinary clinical practice and biomedical research introduces changes to veterinary care by boosting diagnostic abilities as well as improving resistant bacteria tracking while revealing antibiotic utilization patterns (1). AI solutions successfully predict resistance potentials in particular pathogens to determine optimal antimicrobial treatments which leads to better outcomes and improved antimicrobial stewardship practices.

The review by Caneschi et al. (4) demonstrates how machine learning techniques function for antimicrobial resistance prediction through their assessment of ML applications for AMR prediction and treatment selection guidance and resistance monitoring (2). The literature review conducted by Sakagianni et al. (5) presents evidence about machine learning's usage for AMR prediction that supports the creation of clinical decision support systems. Liu et al. (6) in their study present the resistance of Actinobacillus pleuropneumoniae to different antimicrobial drugs was predicted through ML models utilizing whole genome sequences.

## 3 AMR dashboard construction methodology

### 3.1 Dataset description

The dataset contains 959 data points gathered in three veterinary practices, located in West Texas. Fourteen-year period from 2011 to 2024. Every dataset is associated with a single diagnostic record. sent to local veterinary diagnostic laboratories. Such records are real-world, antimicrobial susceptibility testing (AST) results obtained using clinical samples of companion, animals (dogs, cats, horses), livestock (cattle, sheep, goats), and recently rare species (rabbits, deer, and exotic animals). Each record in the dataset includes the following data elements:

- **Animal host species:** The originating species of the clinical sample (e.g., dog, horse, cat, cattle).
- **Sample collection date:** The date when the sample was submitted and processed, enabling chronological trend analysis.
- **Bacterial isolate:** The species or subspecies of bacteria identified from the sample (e.g., *Escherichia coli, Staphylococcus pseudintermedius, Streptococcus equi*).
- **Antimicrobial susceptibility test (AST) results:** Minimum inhibitory concentration (MIC) values across a standardized panel of antimicrobials, as well as the interpreted susceptibility categories (Susceptible, Intermediate, Resistant).
- **Antibiotic class and agent:** The drug name and its pharmacological class (e.g., β-lactams, aminoglycosides, fluoroquinolones).
- **Resistance phenotype:** Binary or categorical classification indicating whether the isolate was resistant, intermediate, or susceptible to the tested drug.
- **Metadata:** Additional attributes such as sample source (urine, wound, respiratory), laboratory identifiers, and geographic origin of the veterinary practice.

**Figure 1:**
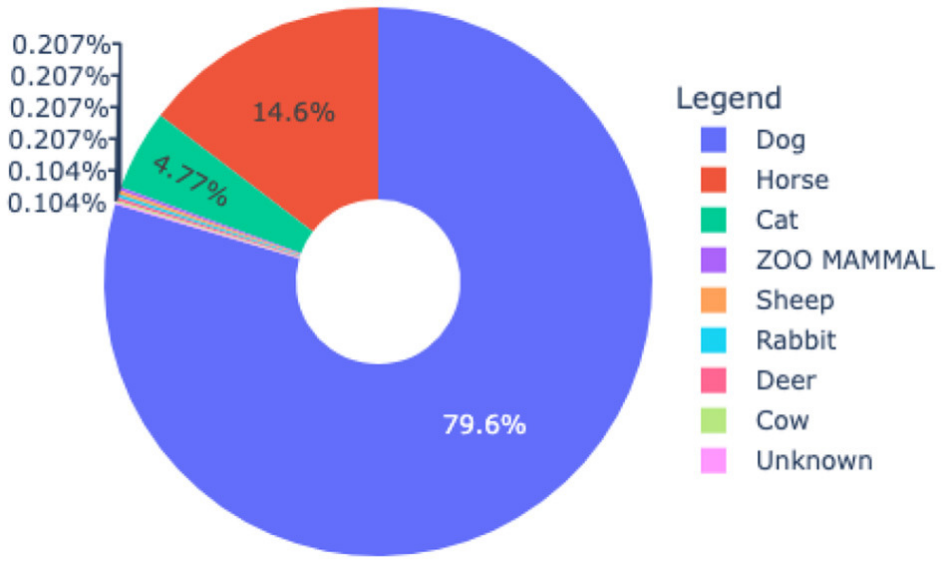
Data distribution based on animals for the AMR dashboard.

Figure 1 presents the data distribution where the dogs have the highest percentage of 79.6% followed by Horses 14.6% and cats 4.77%. The animals such as Zoo mammals, sheep, rabbits and deer each have a percentage of 0.207%. The animals cows and unknown have the lowest percentage of 0.104%. For the experiment, 959 data points were retrieved from three veterinary practices from West Texas spanning 14 years period (2011–2024).

The dataset was thoroughly preprocessed to guarantee its dependability and quality. The first steps in preprocessing actions included converting the date column to DateTime format and sorting by date sequence while locating missing values. The analysis of numerical columns demanded an advanced technique because these values served essential functions in trend analysis accuracy. The sequential nature of data made it possible to implement the *Recurrent Neural Network (RNN)* model for missing numeric value imputation. An RNN used Masked Layers to bypass lacking values in its training process while learning historical trends for point reconstruction. RNN-based imputation stands superior to standard statistical completion methods for time-oriented datasets since it learns natural temporal relationships.

In this experiment to deal with missing data, the Recurrent Neural Network (RNN) model design includes two sequential SimpleRNN layers having 64 and 32 hidden units with ReLU activation. A dense layer in the model performs a reconstruction of missing values throughout all features. The hyperparameter tuning values were chosen throughout the observation during the experiment. The model utilize adam (7) as an optimizer and MSE as a loss function during the training. The model was trained with 50 epochs with 16 batch size to achieve effective learning and prevent overfitting. The models were trained on the Apple M2 Max studio with 64 GB of memory, utilizing of GPU.

### 3.2 Trend prediction using AI

During the experiment, LSTM and Prophet were chosen intentionally because of LSTM captures complex temporal dependencies and long-range patterns in time series data and Prophet helps by decomposing trends and seasons in a way that handles skipped and missing values. The models performed better and were more stable than traditional statistical approaches like ARIMA (8), which failed to generalize in the presence of non-linearity and data sparsity.

In order to test the models, we employed walk-forward cross-validation to maintain the time-dependence of the data and avoid look-ahead bias. In this method, the training window is extended one step at a time, the model is trained on data until time *t*, tested on *t*+1 to *t*+*h* (where *h* is the forecast horizon) and then one rolls forward the window. This is continued five times, where the training and test sets are modified dynamically depending on the length of the data. The final outcomes are averaged over 5 folds to make the results more robust. In the case of Prophet, we applied manual walk-forward validation and the test size was estimated as the length divided by the number of splits and one, where the temporal order is required. In the case of LSTM we used manual walk-forward validation as well (early stopping, patience = 10) so that we can avoid overfitting. This validation technique provides good out-of-sample performance estimates, which is not the case with standard k-fold cross-validation, which shuffles data and breaks time dependencies.

#### 3.2.1 Prophet model

Meta previously known as Facebook has created *Prophet* as an additive regression model which serves as an interpretable solution for time series forecasting purposes (9). The model efficiently handles seasonal patterns and trend shifts along with underlying pattern changes because of its specific suitability with a commercial focus. Prophet analyzes time series data to divide it into trend patterns and seasonal components and holiday effects before it identifies specific trend change points.

Analysis of antimicrobial resistance trends were performed using forecasting methods based on time series data through the Prophet model. The dataset underwent split division (80% training and 20% testing) while the model received configuration parameters including multiplicative seasonality with a changepoint prior scale value of 0.05 and yearly, weekly and customized monthly seasonality. After training, future data generation through prediction enabled monitoring of resistance trends to create intervention strategies.

#### 3.2.2 Long Short-Term Memory (LSTM)

The Long Short-Term Memory (LSTM) model (10) served as the data processing method because it functioned as a specialized recurrent neural network (RNN) to detect temporal dependencies and sequential patterns in the data. The gated architecture of LSTM networks provides superior time series modeling capabilities because it maintains long-term data while overcoming gradient vanishing and exploding problems. The LSTM memory cell consists of an input gate, output gate and forget gate. The input gate determines the volume of new input which will become part of the cell state. The forget gate determines what information should be removed from the cell state. Through the output gate LSTM determines which information from the cell state will be transmitted to the following time interval. The gates inside LSTMs enable their ability to preserve vital information across time which proves useful when performing time series prediction and language processing.

In this study, to train and evaluate the LSTM model the dataset split 80:20 ratio to use its training section and testing section. The training data consisted of 80% of the data whereas testing data used 20% of the dataset. The LSTM network works with sequential data therefore the input features are transformed into three dimensions consisting of sample, time step and feature. The data reshaped to meet the input requirements of the LSTM layer.

The LSTM layer consists of 50 units with a lookback or historical data of 10. The model used the Adam optimizer and MSE loss function during training. An early stopping technique with 10 epochs of patience was used to eliminate overfitting and enhance model capability. The model trains with epochs of 10 with a batch size of 16, while maintaining 20% of training data for validation purposes.

### 3.3 Utility, security, and privacy tradeoff

The fingerprinting technique to protect data needs balancing the conflicting requirements of data privacy and utility. Our privacy framework uses the parameter ϵ to protect data privacy yet the smaller the value of ϵ leads to higher accuracy. Data accuracy risk decreases when decision models heavily depend on unchanged datasets. Higher data accuracy standards mean that modifications to the records should remain minimal thus requiring greater ϵ values for providing less stringent privacy guarantees. Hence, the design of the fingerprinting mechanism should constructed considering the tradeoff between preserving utility and ensuring security. The use of random steganographic fingerprinting enables direct alignment between established privacy proofing and sustainable utility levels to maintain accurate analysis results. Our approach maintain to achieve high data quality for decision support while overseeing both the privacy requirements and the need for data quality.

Our methods unite to build an operational data sharing system which satisfies legal restrictions and business demands. The fingerprinting process for distributed copies provides tracking abilities for locating unauthorized redistribution activities to their origins. The combination of optimized ϵ and data accuracy levels provides the system with strong analytical capabilities for applications requiring high performance together with strong privacy standards.

As demonstrated in our prior work (11), an ϵ-entry-level differentially-private fingerprinting mechanism was proposed based on a bit-level random response scheme. Although randomness introduces variability in outputs, each service provider (SP) can receive a *unique and reproducible* fingerprinted database based on its public key.

To ensure this, the database owner first collects all fingerprintable bits (i.e., the last *K* insignificant bits of each attribute) into a set: *P* = {*r*_*i, t, k*_|*i*∈[1, *N*], *t*∈[1, *T*], *k*∈[1, min(*K, K*_*t*_)]}, where *N* is the number of records, *T* is the number of attributes, and *K*_*t*_ is the bit-length of attribute *t*. For each SP with external identity *ID*_external_, the owner generates an internal identity *ID*_internal_ and a unique fingerprint: *f* = HMAC_*Y*_(*ID*_internal_), where *Y* is the secret key of the database owner and HMAC is a cryptographic hash-based MAC. Let *L* denote the fingerprint length.

A pseudorandom generator *U* (seeded with *s* = *Y*||*r*_*i, t, k*_) selects which bits in *P* to modify. For each selected position, the owner computes a mask bit *x* and assigns a fingerprint bit *f*∈{0, 1}. The marked bit is given by *B* = *x*⊕*f* with flipping probability parameterized by a calibrated Bernoulli distribution to ensure ϵ-differential privacy at the entry level. This construction guarantees (i) uniqueness of each SP's fingerprinted copy, (ii) reproducibility by the data owner, and (iii) a provable privacy-utility tradeoff.

## 4 Results

### 4.1 Analysis of deep learning prediction

#### 4.1.1 Mean Absolute Error (MAE) value—Prophet model

**Figure 2:**
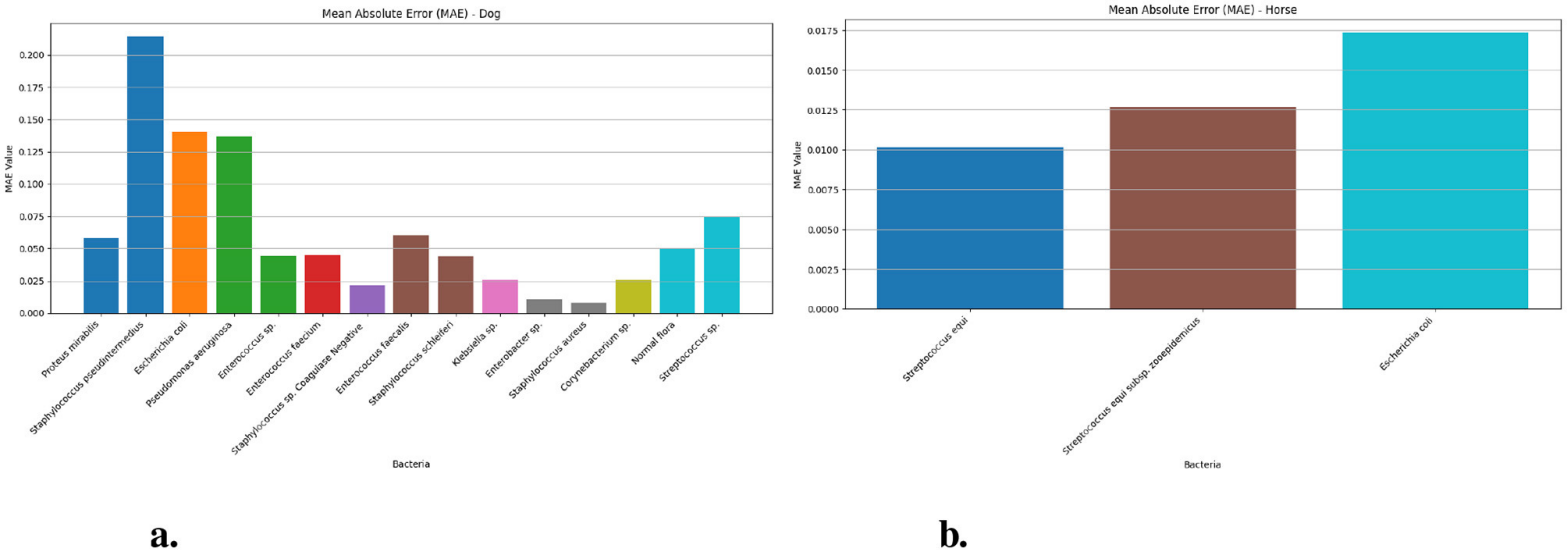
The MAE values for different bacteria in dog and horse samples—Prophet model. **(a)** MAE values for different bacterial species in dog samples. **(b)** MAE values for different bacterial species in horse samples.

Figure 2 presents the Mean Absolute Error (MAE) values of the Prophet model in dog and horse samples for various bacterial species. For the horse samples (Figure 2b), MAE measurements obtained for testing *Streptococcus equi* came to 0.010 (95% CI: 0.008–0.01) and *Streptococcus equi* subsp. zooepidemicus generated 0.012 (95% CI: 0.009–0.02) while *Escherichia coli* yielded 0.017 (95% CI: 0.02–0.02). In comparison, the dog samples (Figure 2a) achieved the highest MAE value of 0.213 (95% CI: 0.20–0.23) for *Staphylococcus pseudintermedius* and the lowest of 0.008 (95% CI: 0.005–0.01) for *Staphylococcus aureus*. Additional MAE for bacterial species isolated from dog samples spanned from 0.010 for *Enterobacter* sp. to 0.140 for *Escherichia coli* with *Enterococcus* sp., *Enterococcus faecium* and *Enterococcus faecalis* having values of 0.044, 0.045, and 0.060.

#### 4.1.2 Root Mean Squared Error (RMSE) value— Prophet model

**Figure 3:**
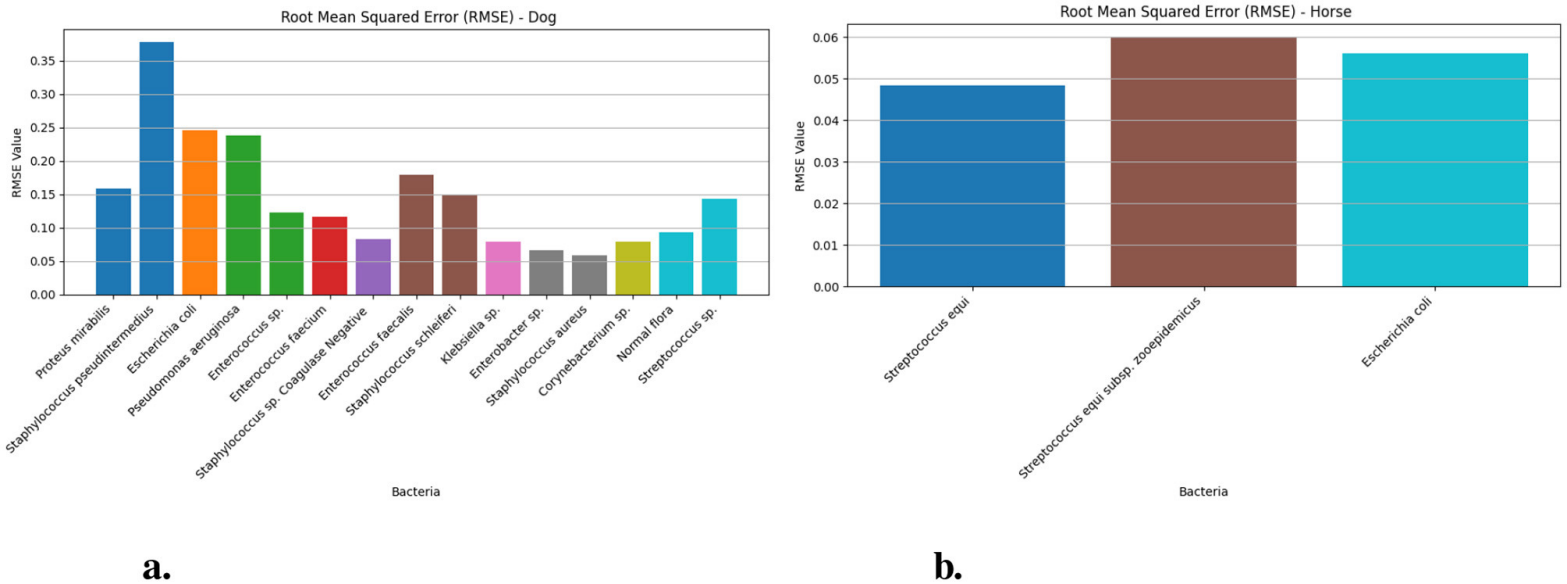
The RMSE values for different bacteria in dog and horse samples—Prophet model. **(a)** RMSE values for different bacterial species in dog samples. **(b)** RMSE values for different bacterial species in horse samples.

Figure 3 demonstrates the Root Mean Squared Error measurement values of dog and horse samples from the Prophet model for different bacteria. The RMSE for *Streptococcus equi* in horse samples (Figure 3b) yielded 0.048 (95% CI: 0.03–0.06) and *Streptococcus equi* subsp. zooepidemicus generated 0.060 (95% CI: 0.06–0.1) and *Escherichia coli* produced 0.056 (95% CI: 0.06–0.09). The RMSE (Figure 3a) analysis showed *Staphylococcus pseudintermedius* reaching its maximum value of 0.377 (95% CI: 0.37–0.43) in dog samples but *Staphylococcus aureus* exhibited the lowest value of 0.058 (95% CI: 0.05–0.08) within the same group. Other notable RMSE values included 0.245 for *Escherichia coli* and 0.158 for *Proteus mirabilis* with *Pseudomonas aeruginosa* at 0.237 and *Enterococcus faecalis* at 0.178.

#### 4.1.3 Mean Absolute Error (MAE) value—LSTM model

**Figure 4:**
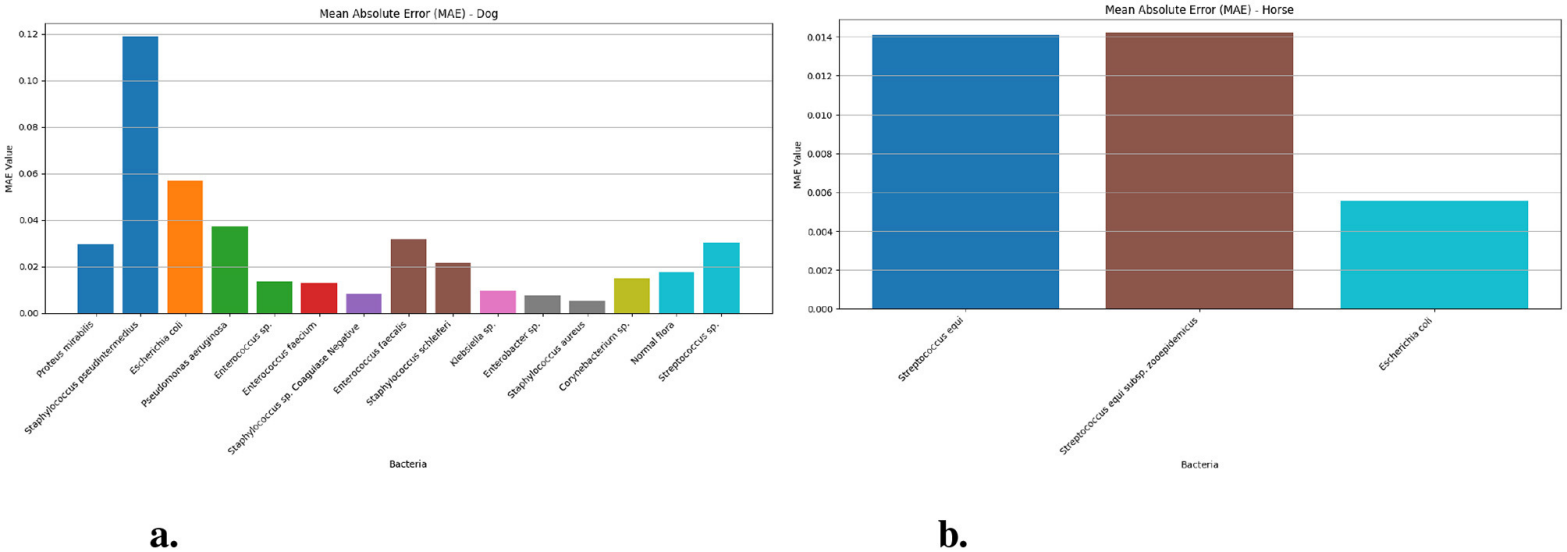
The MAE values for different bacteria in dog and horse samples—LSTM model. **(a)** MAE values for different bacterial species in dog samples. **(b)** MAE values for different bacterial species in horse samples.

Figure 4 results through Mean Absolute Error (MAE) measurements in dogs and horses. Figure 4a prediction of *Staphylococcus pseudintermedius* cases in dogs faces the most difficulty with an MAE value of 0.118 (95% CI: 0.11–0.13) while Escherichia coli comes second at 0.130 (95% CI: 0.05–0.07) and *Pseudomonas aeruginosa* 0.037 (95% CI: 0.03–0.05). The Mean Absolute Error indicators for *Enterococcus faecalis* 0.031 (95% CI: 0.02–0.04), *Proteus mirabilis* stands third at 0.029 (95% CI: 0.02–0.04) and *Staphylococcus schleiferi* (0.021) fall between low and high levels. The LSTM models indicate outstanding accuracy when used to identify *Staphylococcus aureus, Enterobacter* sp., *Staphylococcus* sp. Coagulase Negative and *Klebsiella* sp. since their MAE values are very low at 0.005, 0.007, 0.008, and 0.009 respectively. LSTM Model predictions for horse-based (Figure 4b) bacteria achieve lower MAEs throughout while *Streptococcus equi* reports 0.014 (95% CI: 0.01–0.02), Escherichia coli produces 0.005 (95% CI: 0.003–0.007) and *Streptococcus equi* subsp. zooepidemicus delivers 0.014 (95% CI: 0.01–0.02) because the model efficiently predicts equine bacterial specimens.

#### 4.1.4 Root Mean Squared Error (RMSE) value—LSTM model

**Figure 5:**
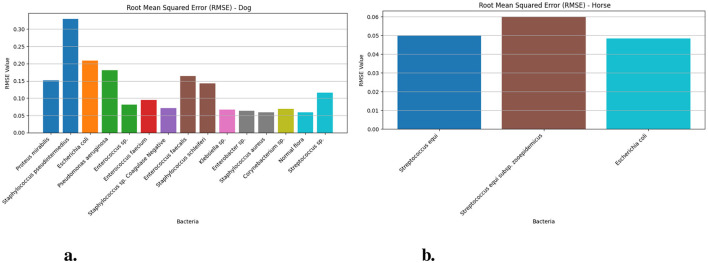
The RMSE values for different bacteria in dog and horse samples—LSTM model. **(a)** RMSE values for different bacterial species in dog samples. **(b)** RMSE values for different bacterial species in horse samples.

Figure 5 demonstrates the Root Mean Square Error (RMSE) values of dog and horse. The Figure 5a presents bacterial species *Staphylococcus pseudintermedius* achieved the highest values of 0.329 (95% CI: 0.30–0.37) while *Escherichia coli* 0.209 (95% CI: 0.20–0.25) and *Pseudomonas aeruginosa* 0.181 (95% CI: 0.16–0.22) show increased deviation from actual values when measured against the dog-related bacteria. *Proteus mirabilis* and *Enterococcus faecalis* also display moderate RMSEs at 0.152 and 0.164, respectively. The accuracy of the LSTM model proves superior for Normal flora (0.058) and two other species *Staphylococcus* sp. Coagulase Negative (0.071) and *Klebsiella* sp. (0.066) because of their low prediction errors. RMS error values in horses (Figure 4b) stay stable for all bacterial groups because *Streptococcus equi* shows 0.049 (95% CI: 0.04–0.07), *Escherichia coli* shows 0.048 (95% CI: 0.05–0.08) and *Streptococcus equi* subsp. zooepidemicus exhibits 0.060 (95% CI: 0.06–0.09) which proves that predictions from equine (i.e., Horse) samples maintain higher stability and accuracy than predictions from canine (i.e., Dog) data.

### 4.2 Visualization outputs of AMR dashboard

**Figure 6:**
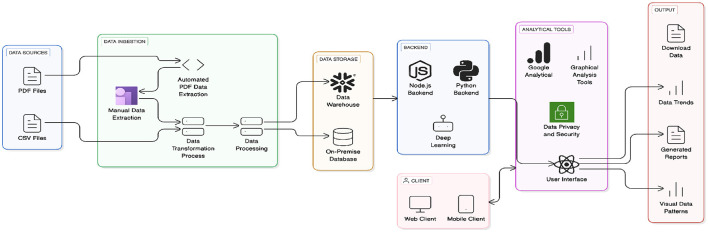
End-to-end data pipeline architecture of AMR dashboard.

Figure 6 the architecture demonstrates the complete workflow from data ingestion (PDF/CSV sources) through data transformation, processing, and storage (data warehouse/on-premise database), supported by a backend (Node.js, Python, deep learning). Analytical tools, including Google Analytics and graphical analysis, ensure data privacy and security. The user interface, accessible via web and mobile clients, delivers outputs like data trends, generated reports, and visual data patterns.

#### 4.2.1 Sankey plot

**Figure 7:**
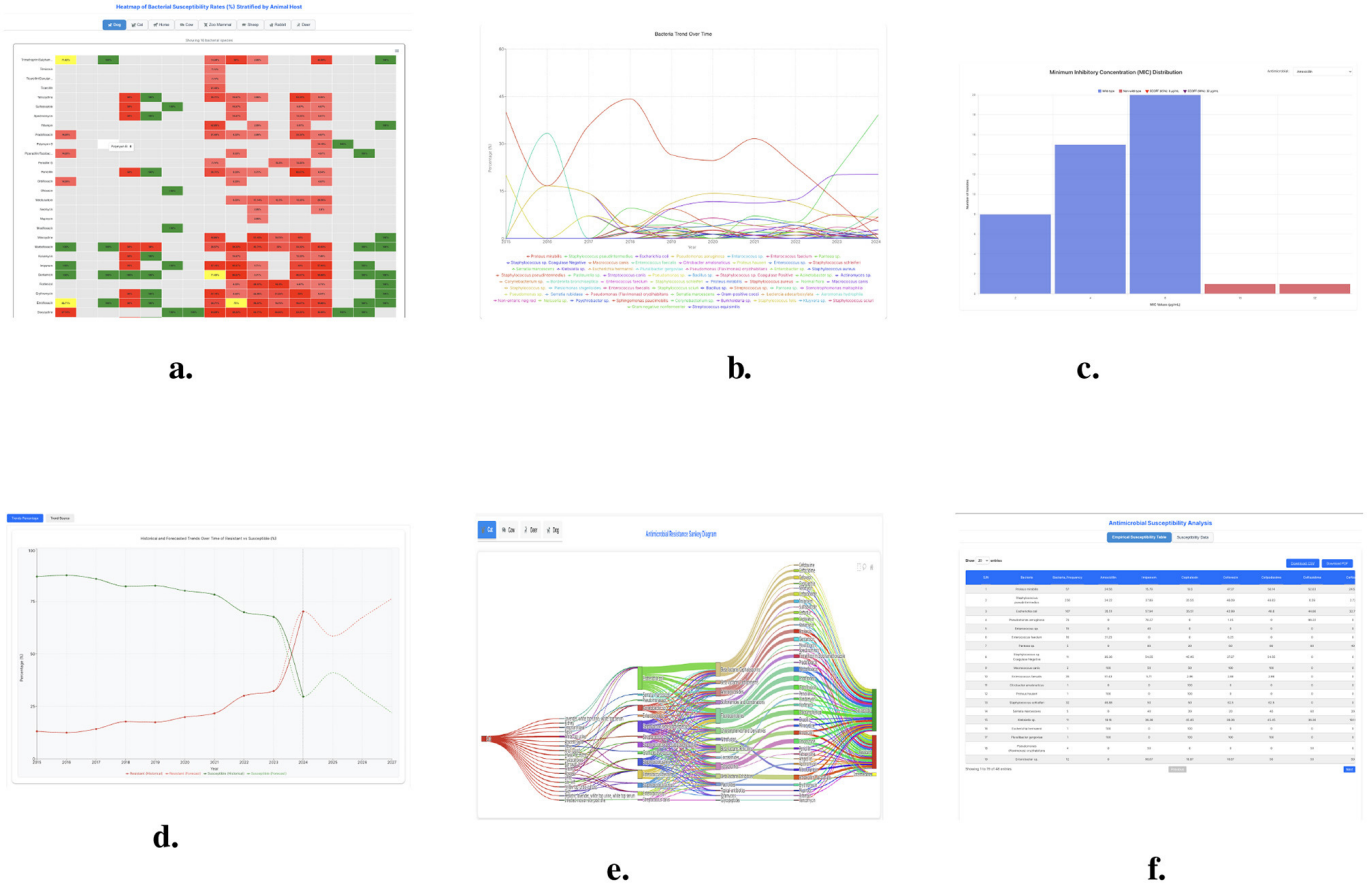
The features of AMR dashboards including (trends, tables, heatmap, Sankey plots, and bar chart). **(a)** Heatmap of bacterial susceptibility rates (%) stratified by animal host. **(b)** Bacterial distribution over year (2015–2024) in (%). **(c)** MIC distribution by along with ECOFF of 95 (%) and 99 (%). **(d)** Historical and forecasted trends over time of resistant vs. susceptible (%). **(e)** Sankey plots by animal host. **(f)** Susceptibility analysis.

The flow diagram displays (12) quantities through arrows which represent proportional flows between sectors using visual representations. These arrows display flow magnitude through their widths and help people understand intricate systems and their relations easily. In our dashboard a Sankey plot (Figure 7e) demonstrates antimicrobial resistance (AMR) trends in bacterial isolates and traces the inter-linked relating: sample sources, bacterial species, antibiotic classes, and response.

#### 4.2.2 Heatmap plot

A heatmap is an image rendering from matrix data where values are visualized as colors having different magnitudes. It facilitates an easy view into patterns, trends, and connections inside massive information sets. Heatmaps are especially helpful when one must deal with big data, which is encoded with various numbers, a color gradient is used, which makes it easier to find a cluster, tied anomalies, and a dependence between variables. The success of a heat map is very much determined by a coupling of color schemes and data normalization. The heatmap (Figure 7a) displays antibiotic resistance trends across antibiotic-susceptibility bacteria pathogens, highlighting the growing challenge of AMR. A color-coding system was used to identify various levels of susceptibility Green for greater than 90% susceptibility, Red for less than 70% susceptibility, and Yellow for intermediate susceptibility (about 70–89 %).

#### 4.2.3 Bacteria distribution

Figure 7b dashboard presents comprehensive changes in bacteria percentages through time to track bacterial population. The dashboard tracks time trends in different bacteria percentages to show changes thus identifying new emerging threats in bacteria. Bering trends emerge in bacterial populations because specific bacteria increase their numbers because antibiotics lose their effectiveness. The bacterial percentage trends reveal essential findings about swift resistant strain expansion and recently introduced resistant bacteria as well as how regional healthcare measures impact antimicrobial resistance. The dashboard helps antimicrobial resistance management by analyzing trends that support veterinary to make better decisions through informed assessment of patient outcomes.

#### 4.2.4 Minimum Inhibitory Concentration (MIC) distribution

The Figure 7c Minimum Inhibitory Concentration (MIC) distribution graph displays bacterial isolate susceptibility patterns. The frequency count divides bacterial isolates between susceptible wild-type strains and non-wild-type strains resistant using ECOFF (Epidemiological Cutoff Values) markers to indicate resistance levels. Most bacterial isolates demonstrate Minimum Inhibitory Concentration values between 2 and 8 μg/mL which is considered wild-type susceptibility but 16 μg/mL or higher MICs identify non-wild-type resistance. The Isolates above the ECOFF (99%) of 32 μg/mL and the ECOFF (95%) of 8 μg/mL indicate the emergence of resistance, which calls for ongoing monitoring and modifications to treatment regimens.

### 4.3 Susceptibility analysis table

The antimicrobial susceptibility table (Figure 7f) effectively shows detailed information about bacterial species together with antibiotic susceptibility patterns. The table shows bacterial occurrences along with percentage sensitivity levels for several antimicrobial drugs that reveal diverse resistance traits among different bacterial species. The susceptibility data demonstrates *Proteus mirabilis* and *Staphylococcus pseudintermedius* demonstrate moderate response to Amoxicillin while *Pseudomonas aeruginosa* displays high resistance properties of multidrug-resistant pathogens. Rates of antibiotic resistance detected in *Enterococcus faecium* remain consistently low for multiple antimicrobial agents which signals major wellness issues in treatment operations. The table reveals the necessity of using specific antimicrobial techniques to deal with infections effectively while resisting microbial resistance.

### 4.4 Historical and forecasted trends of resistant vs. susceptible bacteria

Figure 7d shows that bacterial resistance shows a concerning rise while susceptible bacterial populations decrease. Historical records from 2015 to 2023 document how bacterial susceptible populations have decreased systematically while resistant bacterial strains have grown over time. The prediction deep learning model (i.e., Prophet) predicts that resistant bacterial strains will become greater in number than susceptible bacterial strains after the year 2024.

## 5 Veterinary insights

The AI-driven AMR dashboard developed in this study gives veterinarians an advanced solution that enhances their decision-making ability and improves their veterinary work, especially within Texas' limited-resource rural and regional communities. An AI dashboard employs deep learning models including LSTM and Prophet with Recurrent Neural Networks (RNN) for data imputation to present a collection of expertly crafted features like Sankey plots, bacteria distribution trends, heatmaps and susceptibility tables and comprehensive reports that fulfill veterinary professional requirements. The set of components provides veterinarians with evidence-based direction to manage antimicrobial resistance issues (AMR) while achieving maximum patient results.

Antimicrobial susceptibility evidence-based data available through the dashboard remains a crucial means by which it supports veterinary medical determination-making. Veterinarians can access clear antimicrobial treatment recommendations in the susceptibility tables using data from their own region regarding bacterial resistance. Through consulting the management table a rural veterinarian can pick a suitable empirical treatment for bacterial infections in livestock while both minimizing uncertainty and reducing the chance of administering ineffective medications.

The bacteria distribution trends and heatmaps which present resistant pathogen prevalence and geographic spread across time periods. The Prophet model drives the creation of these trends that help veterinarians detect upcoming bacteria patterns within their practice areas by showing bacteria resistance evolved over time in companion animals. The heatmap provide opportunity for antibiogram that the veterinarians can rely on to make empirical decision. Percentage >90% susceptibility indicates higher probability of therapeutic success while ≤ 70% susceptibility indicates unreliable and unpredictable therapeutic success.Through the heatmap tool a veterinarian can detect resistance clusters which would help them readjust their antimicrobial selection strategy for successful empirical treatments.

In addition, the Sankey plot graph shows how resistance data moves through bacterial species alongside antimicrobials and results. The Sankey plot provides a holistic view and pattern of AMR from clinical samples to different bacterial isolated and resistance development to different antimicrobial classes. From an epidemiological perspective, it may help identify key sources while tracing transmission pathways compared to resistance outbreaks. Further, the visual display may help veterinarians make targeted antimicrobial decisions by revealing both current resistance patterns along with new trends. This information empowers veterinarians in establishing better empirical treatment and infection control methods to reduce antimicrobial resistance.

The dashboard presents complete reports containing both AMR pattern analyses and predictive data and statistical breakdowns to display full detail. The dashboard may be helpful to both animal treatment and government authorities to make strategic decisions through empirical antibiotic treatment evidence. Rural practitioners can use the report to defend adjustments to their treatment plans when seeing livestock farmers or to prepare against incoming resistance risks revealed by predictions. The dashboard enables real-world antimicrobial treatment selection in veterinary clinics for animal patients and helps governmental agencies develop antimicrobial policies through data-based decisions.

## 6 Data sharing for research validation

In the study we utilized the data under user agreements and is subject to intellectual property rights. The data agreements prohibit redistribution or public the datasets. To safeguards proprietary information, our approach to the dashboard requires all data distribution or acquisition from vendors or research needs to be fingerprinted. The fingerprinting approach utilized embedding random steganographic marks into selected insignificant bits of each data point. The random marks represent intrinsic characteristics that allow both data traceability prevention and proof of differential privacy guarantees. The design methodology employs techniques to stop adversaries from correctly reconstructing original data without compromising analytical utility, as demonstrated in prior work (11). The AMR dashboard can be accessed at: https://amrsecuredashboard.com/.

## 7 Conclusion

In this study, we present an AI-based antimicrobial resistance (AMR) dashboard that addresses specific requirements of Texas's rural and regional veterinary practices. The system developed was based on a dataset of the sample size is 959 records of diagnoses made during 14 years (2011–2024) in three West Texas veterinary practices. Each record contained animal host species, bacterial isolates, antimicrobial susceptibility testing (MIC values and resistance categories), and associated metadata, providing a longitudinal view of resistance trends across companion animals and livestock.

By integrating deep learning model such as LSTM and Prophet with RNN-based data imputation techniques the dashboard provides antimicrobial susceptibility trend analysis and forecasting in real-world AMR data. These models were evaluated using mean absolute error (MAE) and root mean squared error (RMSE), demonstrating strong predictive performance. For instance, LSTM achieved lower error rates than Prophet in forecasting resistance for *Staphylococcus pseudintermedius* and *Escherichia coli* in dog samples, while predictions for equine bacterial isolates maintained stable accuracy across both models.

The results highlighted longitudinal patterns: resistant bacterial populations steadily increased from 2015 to 2023, with forecasts predicting resistant strains to surpass susceptible ones after 2024.The study demonstrates that combining robust data analytics with visualization features which include Sankey plots, heatmaps and MIC distribution charts significantly improves the interpretability for veterinary practitioners. The combination of visual aids alongside forecasting components and susceptibility tables provides actionable insights that enable antimicrobial decision-making and antimicrobial stewardship.

The proposed dashboard addresses several pressing gaps in current veterinary AMR data analysis. Traditional statistics fail to adequately manage both high-dimensional datasets as well as time-related information in settings with limited resources. The system application of AI methodologies delivers improved prediction performance as well as recognizes evolving resistance trends. The methodology includes privacy-protection through data fingerprinting with steganographic fingerprinting that uses utility-privacy tradeoff tuning for defending sensitive veterinary data during collaborative research and data sharing.

This research introduces a complete method that both enhances treatment precision and supports disease monitoring and policy formulation. The dashboard enables veterinarians to rapidly detect resistance threats while allowing them to select treatments from regional patterns and achieve optimal antibiotic use confidently. The model provides public health stakeholders with a reusable framework supported by One Health principles that demonstrate the interconnectedness of human, animal and environmental health.

The future work in research development aims to upgrade dashboard functionality through federated learning methods that will enable multi-institutional data analysis while ensuring privacy protection. Technical improvements combining real-time diagnostic capabilities with genomic data integration will boost prediction capabilities making the system relevant for multiple veterinary fields. The redesigned interface will improve accessibility for different user types including both farmers and animal health workers beyond technical professionals. The AMR dashboard represents a fundamental advancement in veterinary healthcare solutions that use data to provide secure and sustainable veterinary healthcare solutions.

## Data Availability

The datasets presented in this article are not readily available due to intellectual property rights. Requests to access the datasets should be directed to the corresponding author.

## Footnotes

1 Access to the AMR data is available upon request, contingent on signing the user agreement required by the data providers.

2
https://amrsecuredashboard.com/
