# Supplementary figures and images for: A data privacy and deep learning based AMR dashboard for rural and regional veterinary practices in Texas
Source: Front Vet Sci. 2025 Dec 10;12:1646675. doi: 10.3389/fvets.2025.1646675 (PMC12739177; doi:10.3389/fvets.2025.1646675)

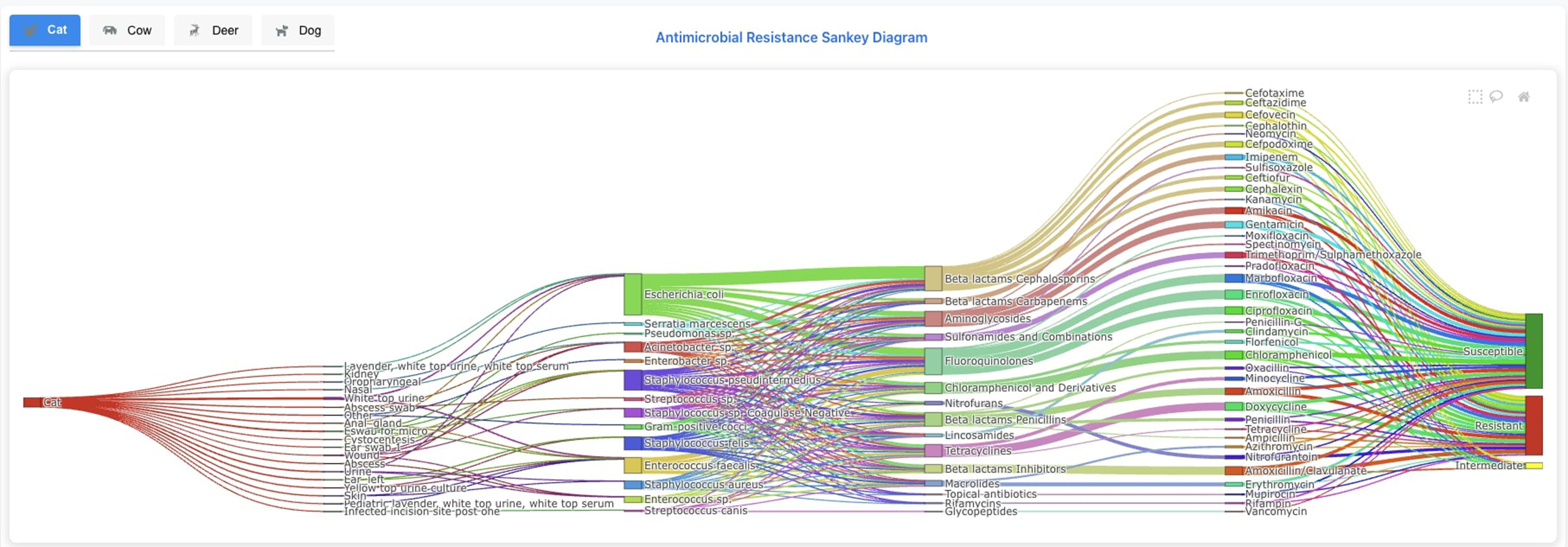

Supplement: Supplementary file 1 [file Image_1.jpeg]
